# Everybody's Page

**Published:** 1892-01-16

**Authors:** 


### EVERYBODY'S PAGE.

"Customers' Milk," as has recently been demonstrated to the British public, is altogether another thing from cow's milk. The latter is to be had, no doubt, for the paying, but the former is not infrequently substituted for it. The substitution of this adulterated commodity is fortunately not always accompanied by profit to the delinquent salesmen. One of these offenders had the questionable pleasure recently of paying a fine of £20 for his playfulness. This ingenious gentleman appears to have carried a can of pure milk in case the inspector should come upon the scene. This the inspector did, and, unfortunately for the offending salesman, proved curious as to the difference between the milk offered him for analysis and the so-called "customers' milk," which proved to be adulterated with water to the extent of one-fourth

It is stated that the Panama Canal Company used two hundred thousand ounces of quinine yearly during the progress of the works. This drug appears to have been the only form of medicine on which any reliance could be placed for the treatment of the various forms of fever in existence on the Isthmus. The most pernicious type of malarial fever almost invariably attacked new comers, but the numerous deaths which occurred were not all due directly to fever. Fright, no doubt, aggravated matters, and many of those employed on the works were in the habit of taking as a preventive a teaspoonful of sulphate of quinine in rum or vermouth every morning on an empty stomach, so that the drug soon became powerless for good, owing to the system becoming inured to its action.

